# Etanercept embedded silk fibroin/pullulan hydrogel enhance cartilage repair in bone marrow stimulation

**DOI:** 10.3389/fbioe.2022.982894

**Published:** 2022-12-08

**Authors:** Xiongbo Song, Xin Wang, Lin Guo, Tao Li, Yang Huang, Junjun Yang, Zhexiong Tang, Zhenlan Fu, Liu Yang, Guangxing Chen, Cheng Chen, Xiaoyuan Gong

**Affiliations:** ^1^ Center for Joint Surgery, Southwest Hospital, Third Military Medical University (Army Medical University), Chongqing, China; ^2^ College of Medical Informatics, Chongqing Medical University, Chongqing, China

**Keywords:** etanercept, silk fibroin, pullulan, cartilage repair, bone marrow stimulation

## Abstract

**Background:** Bone marrow stimulation (BMS) is the most used operative treatment in repairing cartilage defect clinically, but always results in fibrocartilage formation, which is easily worn out and needs second therapy. In this study, we prepared an Etanercept (Ept) embedded silk fibroin/pullulan hydrogel to enhance the therapeutic efficacy of BMS.

**Methods:** Ept was dissolved in silk fibroin (SF)—tyramine substituted carboxymethylated pullulan (PL) solution and enzyme crosslinked to obtain the Ept contained SF/PL hydrogel. The synergistical effect of SF/PL hydrogel and Ept was verified by rabbit osteochondral defect model. The mechanism of Ept in promoting articular cartilage repair was studied on human osteoarthritic chondrocytes (hOACs) and human bone marrow mesenchymal stromal cells (hBMSCs) *in vitro*, respectively.

**Results:** At 4 and 8 weeks after implanting the hydrogel into the osteochondral defect of rabbit, histological analysis revealed that the regenerated tissue in Ept + group had higher cellular density with better texture, and the newly formed hyaline cartilage tissue was seamlessly integrated with adjacent native tissue in the Ept + group. In cellular experiments, Ept treatment significantly promoted both gene and protein expression of type II collagen in hOACs, while decreased the protein levels of metalloproteinase (MMP)-13 and a disintegrin and metalloprotease with thrombospondin motifs 5 (ADAMTS5); alcian blue staining, type II collagen and aggrecan stainings showed that addition of Ept significantly reversed the chondrogenesis inhibition effect of tumor necrosis factor alpha (TNF-α) on hBMSCs.

**Conclusion:** BMS could be augmented by Ept embedded hydrogel, potentially by regulating the catabolic and anabolic dynamics in adjacent chondrocytes and enhancement of BMSCs chondrogenesis.

## Introduction

Osteoarthritis (OA), which associates with the dysfunction of adult articular cartilage, is the most common form of joint disease, and may result in arthralgia, joint deformation, and limited mobility in patients (Quicke et al., 2022). Among several risk factors (e.g. genetics, age, and obesity), articular cartilage injury caused by trauma or disease remains a high risk factor for OA. Due to the avascular and low cellularity nature of articular cartilage, intrinsic repair of defect is remarkably difficult *in vivo*, and surgical intervention is often required (DeFrate et al., 2019). Bone marrow stimulation (BMS) is a technique developed in the 1950s, and has been widely used in clinic to regenerate hyaline cartilage-like tissues from damaged articular cartilage (Makris et al., 2015; Kwon et al., 2019; Sahranavard et al., 2022). During the procedure, a physical conduit is created to connect vascularized subchondral bone marrow with the debrided cartilage lesion. Bone marrow substrates, including bone marrow mesenchymal stromal cells (BMSCs), growth factors, and cytokines are introduced into damaged articular cartilage, participating in the repair process. However, due to the inflammatory micro-environment of injured articular cavity, and insufficient mechanical support of bone marrow effusion, studies reported mechanically inferior collagen I/II fibrocartilage as the main outcome of BMS (Mithoefer et al., 2009).

Once injured, cartilage fragments are released into articular cavity, leading to the activation of immune cells such as macrophages and T cells, producing interleukin-1β (IL-1β) and tumor necrosis factor alpha (TNF-α) (Sharma et al., 2020). These inflammatory factors create chronic inflammatory micro-environment, and react upon chondrocytes to secrete metalloproteinases (MMPs) and a disintegrin and metalloprotease with thrombospondin motifs (ADAMTS), accelerating the degradation of type II collagen and aggrecans in cartilage matrix (Wang and He, 2018). Etanercept (Ept) is a recombinant soluble p75 TNF receptor, which has high affinity for TNF-α, preventing it from binding with its receptor (Garrison and McDonnell, 1999). Clinically, Ept is used as disease-modifying anti-rheumatic drugs (DMARDs), which could significantly alter disease progression (Smolen et al., 2017). In addition to the above descripted indication, an *in vivo* study demonstrated that subcutaneous injection of Ept promoted repair of osteochondral defects in the rabbit model (Kawaguchi et al., 2009). Application of the anti-TNF-α monoclonal antibody demonstrated reversal of cartilage degradation in polyarthritic TNF-α-overexpression mice (Shealy et al., 2002), indicating that Ept could serve as an inflammatory regulator to strengthen BMS in cartilage repair.

In addition to chemical environment, proper physical environments are required for migration, adhesion, and cartilaginous differentiation of BMSCs. The blood clot forms at the defect site post BMS operation is mainly composed of BMSCs, erythrocytes, leukocytes, and fibrinogen. Considering the poor mechanical property of blood colt, patients are advised to follow strict rehabilitation program to ensure gradual increase in mechanical stimulation postoperatively (Hurst et al., 2010), and stabilization of the clot by incorporating scaffold may improve the ensuing repair response. Recently, acellular bio-scaffolds have been fabricated to augment BMS (Armiento et al., 2018; Armiento et al., 2019). Bio-scaffolds with mechanical property similar to that of native articular cartilage provide suitable physical environment for the ingrowth, proliferation and chondrogenic differentiation of BMSCs. Simultaneously, the inflammatory microenvironment can be regulated by embedding immuno-modulatory elements in the bio-scaffolds (Glass et al., 2014; Tamaddon et al., 2020; Tong et al., 2020). Among different forms of acellular bio-scaffolds, hydrogels with good biocompatibility, proper degradation rates, and tissue-matched elasticity are widely used (Liu et al., 2020; Wagenbrenner et al., 2021; Huang et al., 2022). Previously, we prepared an enzymatically crosslinked silk fibroin/pullulan (SF/PL) hydrogel, and proved its feasibility for musculoskeletal tissue engineering (Li et al., 2018). Here, we assumed that clinical outcome of BMS could be enhanced by combination of SF/PL hydrogel and Ept.

In the present study, to enhance the current therapeutic effect of BMS, SF/PL hydrogel containing Ept was fabricated and performed with BMS. The synergistical effect of SF/PL hydrogel and continuously released Ept on articular cartilage repair was verified by rabbit osteochondral defect model. To investigate the mechanism of Ept in enhancing articular cartilage repair, human osteoarthritic chondrocytes (hOACs) and human bone marrow mesenchymal stromal cells (hBMSCs) were employed as *in vitro* model. The effects of Ept on catabolic and anabolic dynamics in hOACs, and on chondrogenetic differentiation in hBMSCs were analyzed, respectively.

## Materials and methods

*Preparation of Ept contained SF/PL hydrogel.* Silk fibroin (SF) and tyramine substituted carboxymethyl pullulan (PL) were synthesized according to our previous study (Li et al., 2018). Ept contained SF/PL hydrogels were prepared by an enzyme-mediated polymerization strategy. In a typical procedure, 40mgEpt was dissolved in 2 ml of SF (30 mg/ml)—PL (6 mg/ml) solutions, the concentration of Ept used was mainly decided by converting equivalent dosage between rabbit and human according to body surface area (Nair et al., 2016; Nair et al., 2018). 10 μl of HRP solution (1000 U/mL) was added to 1 ml of SF and PL mixture solution, then 10 μl of H_2_O_2_ (1% v/v) was added and mixed by gentle pipetting. To obtain a proper size (200 ml mixture solution/hydrogel) for *in vivo* implantation, gel-forming procedure was carried out in a custom-made cylinder mold (3.2 mm in diameter, 4 mm in height). No flow within 1min upon inverting the vial was regarded as the gel state.

*Release curve of the Ept in the hydrogel in vitro.* To quantify the release rate of Ept in the SF/PL hydrogel, the Ept embedded SF/PL Hydrogel was cultured in PBS in dialysis bag (3500 KDa) at 37°C. After culturing for 0.5 days, 1 day, 2 days, 3 days, 4 days and 5 days, the dialysate was collected and equal amount of PBS was supplemented. The content of Ept in the dialysate was detected through bicinchoninic acid (BCA) assay. All release experiments were conducted in triplicates.

*Implantation of hydrogel into osteochondral defect in rabbit knee joint.* All procedures were in accordance with the Guide for the Care and Use of Laboratory Animals, and were approved by the Institutional Animal Care and Use Committee of Third Military Medical University (Army Medical University, AMUWEC20211199). The operations were performed according to our previous study (Wang et al., 2019). Briefly, 18 skeletally mature New Zealand White rabbits (female, 2 kg) were randomly divided into untreated group (NC, *n* = 6), experiment group (Ept+, *n* = 6) and none Ept control group (Ept-, n = 6). Under anesthesia by pentobarbital sodium (50 mg/kg), BMS was simulated by creating osteochondral defect with a sterile electric drill (3.2 mm in diameter, 4 mm in depth) in the femoral trochlear groove of the left hind limb. Visible bleeding was observed to ensure that the defects reached subchondral bone (Figure 2A). For Ept+ and Ept- groups, defects were implanted with Ept contained SF/PL hydrogel (3.2 mm in diameter, 4 mm in height, *n* = 6) and SF/PL hydrogel alone (*n* = 6) respectively. At 4 and 8 weeks post implantation, animals were sacrificed (3 rabbits each time point) for histological observation.

*Gross and histologic evaluation*. For histologic analysis, specimens were fixed in 10% formaldehyde, decalcified in EDTA for 3–4 weeks, dehydrated in a graded ethanol series, and embedded in paraffin. Samples were cut in the sagittal plane into 4 mm-thick sections through the center of the defect. Paraffin sections were subjected to hematoxylin and eosin staining, safranin O staining, and immunohistochemical staining of collagen types I, II, and X. Cartilage regeneration was analyzed semi-quantitatively with the modified International Cartilage Repair Society (ICRS) gross grading scale (Wayne scoring system) and ICRS visual histologic assessment scale by 3 blinded observers (X.S., X.W., T.L.) (Mainil-Varlet et al., 2003; Wayne et al., 2005; Lee et al., 2018).

*Cell culture of hBMSCs and hOACs.* hBMSCs were purchased from American Type Culture Collection (ATCC) and expanded with human mesenchaymal stem cell growth medium (Cyagen, HUXMX-90011). Cells at passage 7 were used in the following experiments. Human osteoarthritic chondrocytes (hOACs) were obtained from primary knee OA patients undergoing total knee replacement after informed consent and approval from the Ethics Committee of Southwest Hospital (Chongqing, China). As previously described (Huang et al., 2017), cartilage specimens were washed in PBS three times and then diced. hOACs from the diced tissues were isolated by digesting the matrix overnight in high-glucose DMEM (Invitrogen) supplemented with 0.2% type II collagenase (Sigma). The resulting cell suspension was filtered through a 40-μm cell strainer; collected cells were centrifuged (1000rpm for 5 min), and resuspended in high-glucose DMEM supplemented with 10% FBS (Ausbian). The medium was changed every 2 days hOACs at passage 0 from three patients were used.

*In vitro Ept concentration screening.* Ept was dissolved in high-glucose DMEM to obtain final concentration of 2.5 μg/ml, 5 μg/ml, 10 μg/ml, 20 μg/ml, and 40 μg/ml hBMSCs or hOACs were seeded on 96-well plates (5× 10^3^ cells/well, n = 6 each dosage) 12 h before the addition of Ept. Cell viability was analyzed with Cell Counting Kit–8 (Beyotime) after 48 h coculture. Equal amount of culture medium was used as control.

*Effects of Ept on hOACs phenotype.* hOACs were seeded on confocal Petri dish (1× 10^5^ cells/dish), 30 mm cell-culture dish (1× 10^6^ cells/dish), 100 mm cell-culture dish (5× 10^6^ cells/dish) and 6-well plates (1× 10^6^ cells/well) for immunofluorescent staining, mRNA expression level, protein expression level, and cytokines expression level detection, respectively. The hOACs cells cultured in high glucose DMEM culture medium served as control group (*n* = 3) and hOACs cultured in high glucose DMEM culture medium supplemented with 20 μg/ml Ept as experimental group (n = 3). After 48 h culture, cells were collected for analysis respectively, and cell culture medium of hOACs at 1, 3 and 7 days were collected for cytokines detection by enzyme-linked immunosorbent assay (ELISA).

*Cartilaginous differentiation of hBMSCs.* hBMSCs were seeded on confocal Petri dish (1× 10^5^ cells/dish), 30 mm cell-culture dish (1× 10^6^ cells/dish), and 100 mm cell-culture dish (5× 10^6^ cells/dish) for immunofluorescent staining, and detection of mRNA expression level (n = 3), alcian blue staining (n = 5), and protein expression level, respectively. The hBMSCs cultured in chondrogenic differentiation medium were served as control group, chondrogenic differentiation medium with 50 ng/ml TNF-α as TNF-α group, chondrogenic differentiation medium with 20 μg/ml Ept as Ept group, chondrogenic differentiation medium with 50 ng/ml TNF-α and 20 μg/ml Ept as TNF-α+ Ept group. The specific culture medium was changed every day. Cells were collected for analysis after 14-day culture.

*Alcian blue staining.* According to the manufacturer’s instruction, the 30 mm cell-culture dishes were washed twice with phosphate buffer saline (PBS) after removing culture medium. Then the cells were fixed with 4% paraformaldehyde (Biosharp) for 20 min followed by triple PBS wash (5 min each wash). Finally, the cells were incubated with alcian blue working solution (Cyagen, Guangzhou, China) for 30 min and washed with tap water for 5 min. Finally, staining was quantified by solubilizing the sample in 6 M guanidine hydrochloride for 8 h at room temperature (RT). The absorbance at 620 nm was measured by spectrophotometry (Varioskan Flash; Thermo Fisher Scientific) (Gong et al., 2019).

*Immunofluorescent staining.* Cells were washed with PBS and fixed with 4% formaldehyde for 20 min at RT. Then, the cells were washed three times with cold PBS and treated with Triton X-100 (Beyotime P0096) for 10 min at RT. The cells were washed again three times with Immunol Staining Wash Buffer (Beyotime P0106) and blocked for 1 h with Immunol Staining Blocking Buffer (Beyotime P0102) at RT. Then hOACs were incubated with Collagen II Antibody (Novus NBP1-77795), Aggrecan Antibody (Novus NB600-504) at the dilution of 1:200, 1:200, respectively, and hBMSCs were incubated with Collagen I Antibody (Novus NB600-408), Collagen II Antibody (Novus NBP1-77795), Collagen X Antibody (Abcam ab182563), Aggrecan Antibody (Novus NB600-504), SOX9 Antibody (Abcam ab185966) at the dilution of 1:200 together, both in Immunol Staining Primary Antibody Dilution Buffer (Beyotime P0103) overnight at 4°C. Next, the cells were washed three times with Immunol Staining Wash Buffer and incubated with relative secondary antibody (Abcam ab150117 and Abcam ab150079) for 1 h at RT and DAPI (Beyotime, C1005) for 10 min at 37°C, washed again and imaged by fluorescence microscopy. To quantify the fluorescence intensity, at least three staining images from each group were analyzed using ImageJ (NIH). The average fluorescence intensity was determined by dividing the corresponding cell area with the optical density (OD).

*Quantitative real-time PCR.* Total RNA was extracted from cells using TRIzol reagent (Invitrogen), and the RNA concentration was determined using a NanoDrop-2000 spectrophotometer (Thermo Scientific). Then, the RNA was reverse transcribed into cDNA using Transcriptor cDNA Synth. Kit 2 (Roche, Basel, Switzerland) according to the manufacturer’s instruction. Quantitative real-time PCR based on FS Essential DNA Green Master (Roche) was performed using primers specific for Col1a1, Col2a1, Col10a1, SOX9, ACAN and GAPDH (Sangon, Shanghai, China). Primer sequences were as follows: Col1a1 forward, 5′-GCG​AGA​GCA​TGA​CCG​ATG​GAT TC-3′, reverse, 5′-GCC​TTC​TTG​AGG​TTG​CCA​GTC​G-3′, Col2a1 forward, 5′-TGC​TGC​CCA​GAT​GGC​TGG​AGG​A-3′, reverse, 5′-TGC​CTT​GAA​ATC​CTT​GAG GCCC-3′, Col10a1 forward, 5′-GCC​ACC​AGG​CAT​TCC​AGG​ATT​C-3′, reverse, 5′-GGA​AGA​CCA​GGC​TCT​CCA​GAG​TG-3′, SOX9 forward, 5′-GAC​TTC​CGC​GAC​GTG​GAC-3′, reverse, 5′-GTTGGGCGGCAG GTACTG-3′ACAN forward, 5′-TCC​TGG​TGT​GGC​TGC​TGT​CC-3′, reverse, 5′-TCTGGCTCG GTGGTGAACTCTAG-3’. Each reaction contained 5 μLcDNA, 10 μLFS Essential DNA Green master mix, 3 μlwater (PCR grade), and 1 μL each of forward and reverse primers (10 μM). Reactions were performed in triplicate. Examination of the melting curve for non-specific peaks were performed to ensure specificity of PCR reactions, and mRNA levels were determined from Ct values according to a previously published method (Pfaffl, 2001). Briefly, the results were analyzed according to the 2−ΔΔ CT method and normalized to the housekeeping gene GAPDH. All data were expressed as mean ± standard deviation (SD) and analyzed by one-way ANOVA. Statistical significance was defined with *p* < 0.05.

*Western blot.* The cells were lysed in RIPA with PMSF (Beyotime, P0013B) on ice for 5 min and removed with a scraper. Then the lysate was centrifuged at 16,000 g for 15 min, and the supernatant was collected. The protein concentration was determined by BCA Protein Assay Kit (Beyotime, P0009). The samples were diluted with SDS-PAGE Sample Loading Buffer (Beyotime, P0015) and kept at 100°C for 10 min 20 μg of protein was loaded in 4–20% SurePAGE, Bis-Tris gels (GenScript, M00655) and run for 30 min at 200 V followed by transferring onto a PVDF membrane at 200 mA. The membrane was washed three times with 2% v/v TBST (tris-buffered saline with Tween-20). Then, the membrane was blocked with Western Blocking Buffer (Beyotime P0023B)for 1 h at RT, washed with TBST, and incubated with mouse monoclonal antibody anti-actin (Santa Cruz,sc-47778), Collagen I Antibody (Novus NB600-408), Collagen II Antibody (Novus NBP1-77795), Collagen X Antibody (Abcam ab182563), Aggrecan Antibody (Novus NB600-504), SOX9 Antibody (Abcam ab185966), MMP-13 Antibody (Novus NBP2-45887), ADAMTS5 Antibody (Novus NBP2-15286) diluted with Primary Antibody Dilution Buffer (Beyotime P0023A) at a dilution of 1:1000, 1:1000, 1:2000, 1:1000, 1:5000, 1:2000, and 1:5000, respectively overnight at 4°C shaker. Next, the membrane was washed four times with TBST for 10 min, incubated with the mouse anti-rabbit IgG-HRP (Santa Cruz, sc-2357, 1:10000), goat anti-mouse IgG-HRP (Santa Cruz, sc-2005,1:10000) for 1 h at RT, washed again four times, and visualized with Western ECL Substrate (Thermo Scientific) for chemiluminescence.

*ELISA.* The collected cell culture medium was centrifuged at 2000g for 15 min, then the supernatant was detected by ADAMTS5 ELISA kit (Cusabio, CSB-EL001312HU) and MMP-13 ELISA kit (Cusabio, CSB-E04674 h) according to the manufacture’s instruction.

*Statistical analysis.* Statistical analyses were performed using Graphpad prism software (GraphPad Software, CA, United States, Version 6). Graphical results were displayed as means ± SD. All data were assessed for normality using the Kolmogorov-Smirnov test and for homoscedasticity using the F-test. The statistical significance differences between Ept+ and Ept- groups were determined by Student *t*-test for parametric data, and by Mann-Whitney test for non-parametric data. The Welch’s correction was applied for variables with unequal variance. The statistical significance differences between multiple groups were determined by One-way ANOVA test and Fisher’s LSD post-test for parametric data, and by Kruskal Wallis test and Dunn’s multiple comparisons post-test for non-parametric data. In all cases, statistical significance was defined with *p* < 0.05.

## Results

### Gross and histologic evaluation

During the 8-week experiment, all surgical incisions in the rabbits healed well without any infection or death. At 4 weeks and 8 weeks after operation, rabbits were sacrificed by carbon dioxide suffocation, and the gross appearance of the joint samples were observed followed by histologic evaluation to assess the effect of Ept in promoting osteochondral defect repair of knee joint.

**FIGURE 1:**
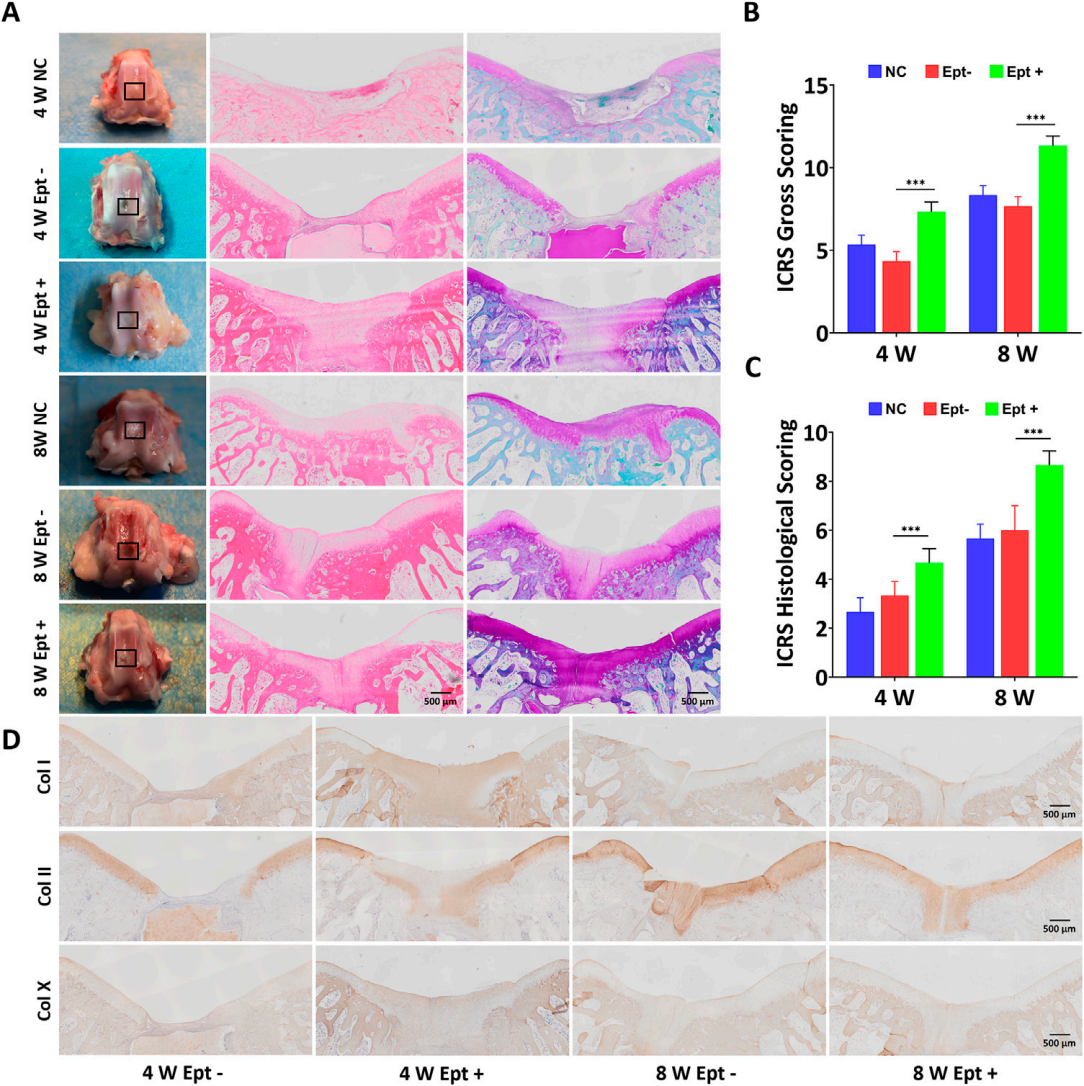
Comparison of osteochondral repair in the untreated group (NC), scaffold-only group (Ept–) and Ept loaded scaffold group (Ept+) at 4 and 8 weeks. **(A)** Macroscopic analysis, hematoxylin and eosin staining, and safranin O staining of the knee joints. Black rectangle indicates the osteochondral defects. **(B)** Macroscopic analysis for cartilage repair with the modified International Cartilage Repair Society (ICRS) gross grading (*n* = 3). **(C)** ICRS visual scoring based on panel D (*n* = 3). **(D)** The immunohistochemical staining against collagen (Col) types I, II and X of the regenerated tissue at 4 and 8 weeks. Mean ± SD.

At 4 weeks postoperatively, the osteochondral defects in the Ept + group were almost completely covered with newly formed tissue while partially were covered in the Ept- group and NC group. At 8 weeks postoperatively, the osteochondral defects in all groups were completely covered with new cartilage tissue. The regenerated tissue in the Ept + group was similar to the surrounding native cartilage, indicating the formation of hyaline cartilage-like tissue, whereas the regenerated tissue in the Ept- group and NC group was irregular and the surrounding tissue was degenerated (Figure 1A). The macroscopic evaluation was confirmed by the averaged ICRS score. Both at 4 weeks and 8 weeks, the ICRS scores of the Ept + group were significantly higher (n = 3, *p* < 0.001) than those of Ept- group (Figure 1B).

Histologic analysis of osteochondral regeneration was carried out *via* hematoxylin and eosin staining and safranin O staining. At 4 weeks postoperatively, both staining showed distinct borders between repaired tissue and surrounding tissue, and the newly formed tissue in Ept + group was thicker than that in Ept- group. At 8 weeks, the surface of the defects became smooth in comparison with that at 4 weeks, and the tissue integration and thickness of Ept + group were superior to that in Ept- group and NC group (Figure 1A). As the ICRS visual histologic assessment showed (Figure 1C), better osteochondral defect repair was observed in Ept + group than that in the other two groups both at 4 weeks (n = 3, *p* < 0.001) and 8 weeks (n = 3, *p* < 0.001).

Qualitative analysis of type II, I, and X collagen expression was conducted upon immunohistochemical staining. No obvious difference in type I and type X collagen expression in regenerated cartilage between Ept+ and Ept- groups at 4 weeks and 8 weeks, as barely positive staining was found. Positive staining of type II collagen in the Ept + group was noticed at 4 weeks, but not in the Ept- group; at 8 weeks postoperatively, both groups showed strong staining for type II collagen with superior deposition and uniform texture in Ept + group (Figure 1D).

**FIGURE 2:**
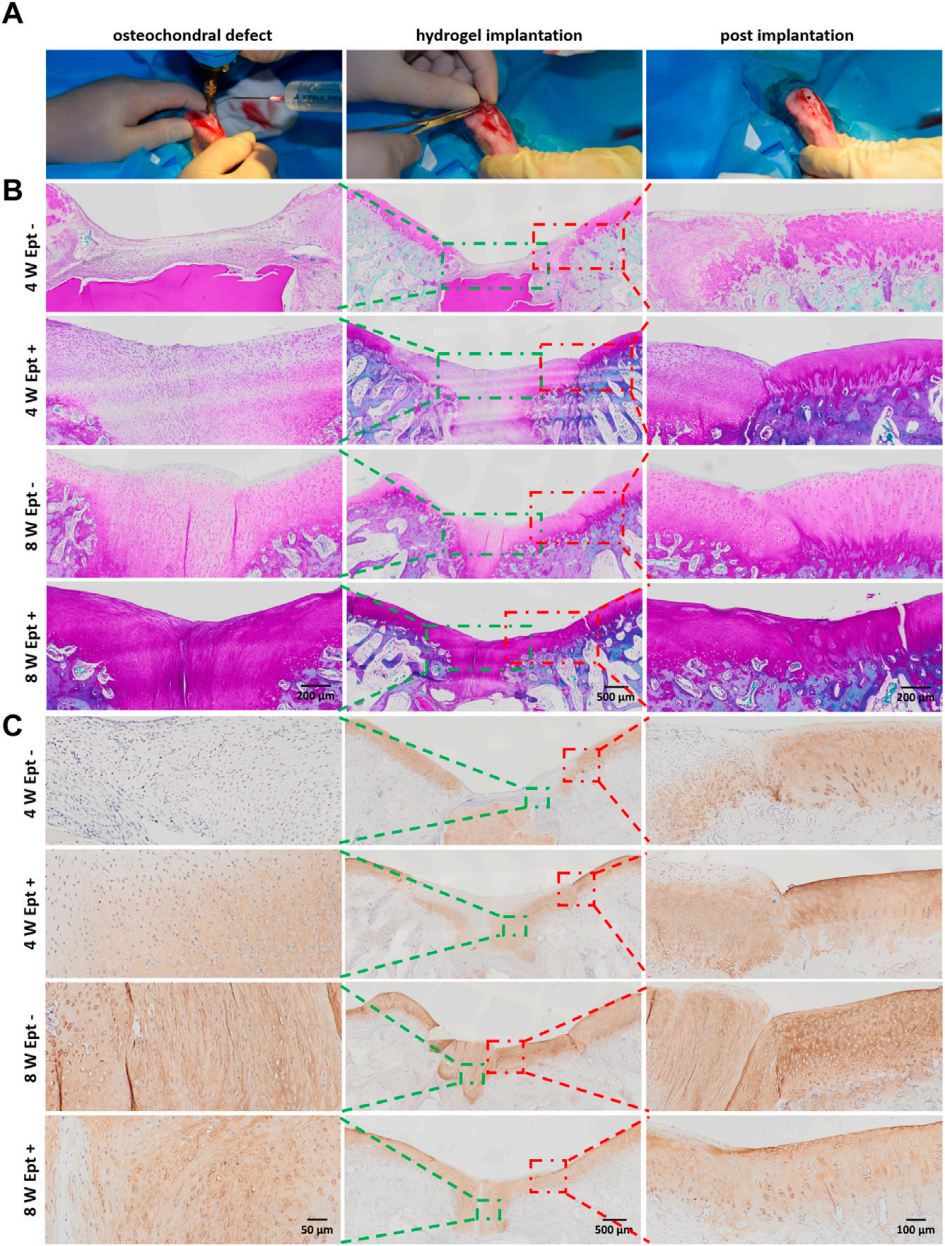
Detailed histological analysis indicated that Ept promoted osteochondral regeneration. **(A)** The creation process of chondral defect in trochlear groove and implantation of SF/PL hydrogel. **(B)** Safranin O staining of the knee joints 4 and 8 weeks postoperatively. **(C)** The immunohistochemical staining against Col II. Green rectangle indicates the center of regenerated tissue, red rectangle indicates the junction between regenerated tissue and adjacent articular cartilage.

To further explore the mechanism of Ept in promoting osteochondral defect repair of rabbit knee joint, detailed differences in cytology and histology were analyzed by safranin O staining and immunohistochemical staining. At 4 weeks postoperatively, compared with Ept- group, intensive cell density in the newly formed cartilage tissue with strong safranin O staining was observed in Ept + group. At 8 weeks postoperatively, significant cartilage matrix deposition was found in the regenerated tissue of Ept + group, and the texture was close to that of native cartilage (Figure 2B, green rectangle). Differences were also found in the junction of regenerated tissue and native cartilage. The boundary between newly formed tissue and native cartilage was obvious in both groups at 4 weeks postoperatively. However, abnormal cellular distribution with weak staining of cartilage matrix in adjacent cartilage of Ept- group was noticed. At 8 weeks postoperatively, compared with distinct boundary in Ept- group, uniform integration was found in Ept + group (Figure 2B, red rectangle). The trend of type II collagen expression was similar to proteoglycan at both defect sites and adjacent cartilage. At both 4 and 8 weeks postoperatively, stronger type II collagen expression was observed in Ept + group than Ept- group at defect sites (Figure 2C).

### Influence of ept on anabolism and catabolism in hOACs

**FIGURE 3:**
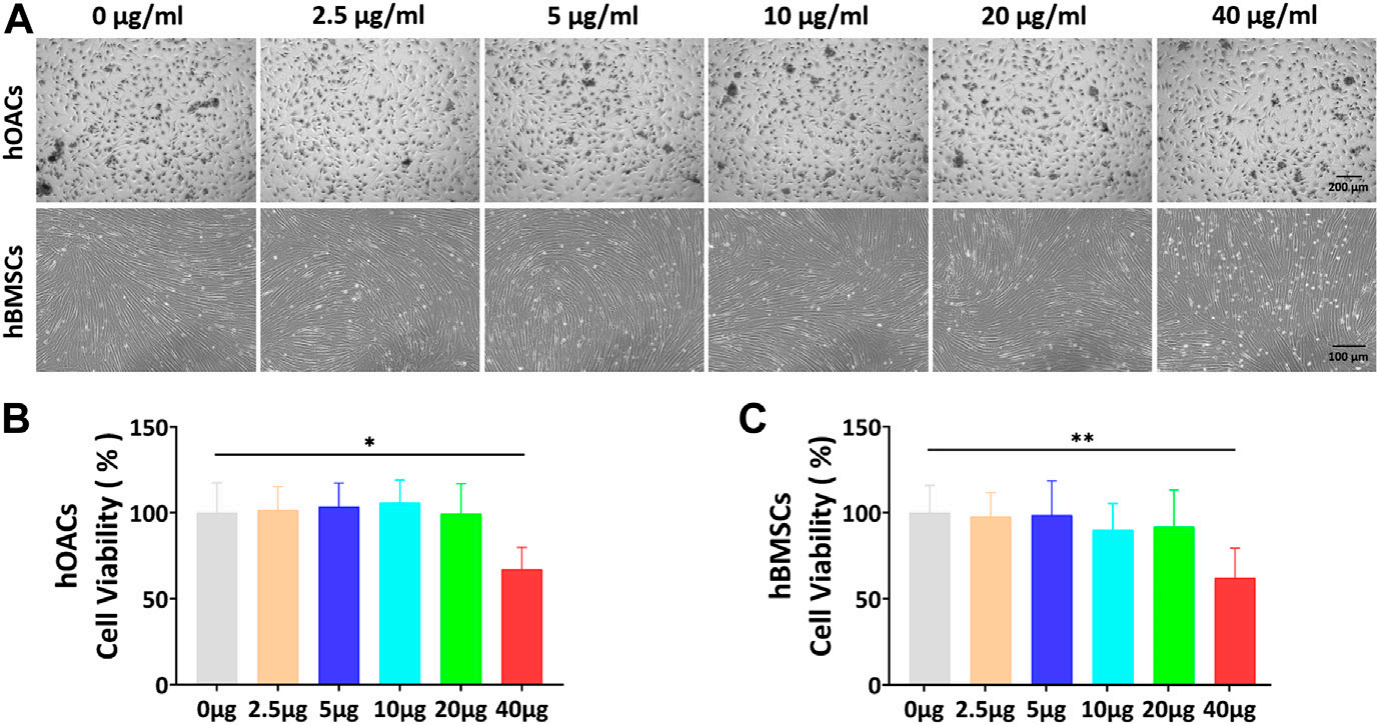
*In vitro* Ept concentration screening for hOACs and hBMSCs treatment. **(A)** Optical microscopy observation of Ept influenced cell viability in hOACs and hBMSCs. **(B and C)** The quantitative analyses of Ept influenced cell viability in hOACs and hBMSCs (*n* = 6). Mean ± SD, *p* ^*^ < 0.05, *p* ^**^ < 0.01.

**FIGURE 4:**
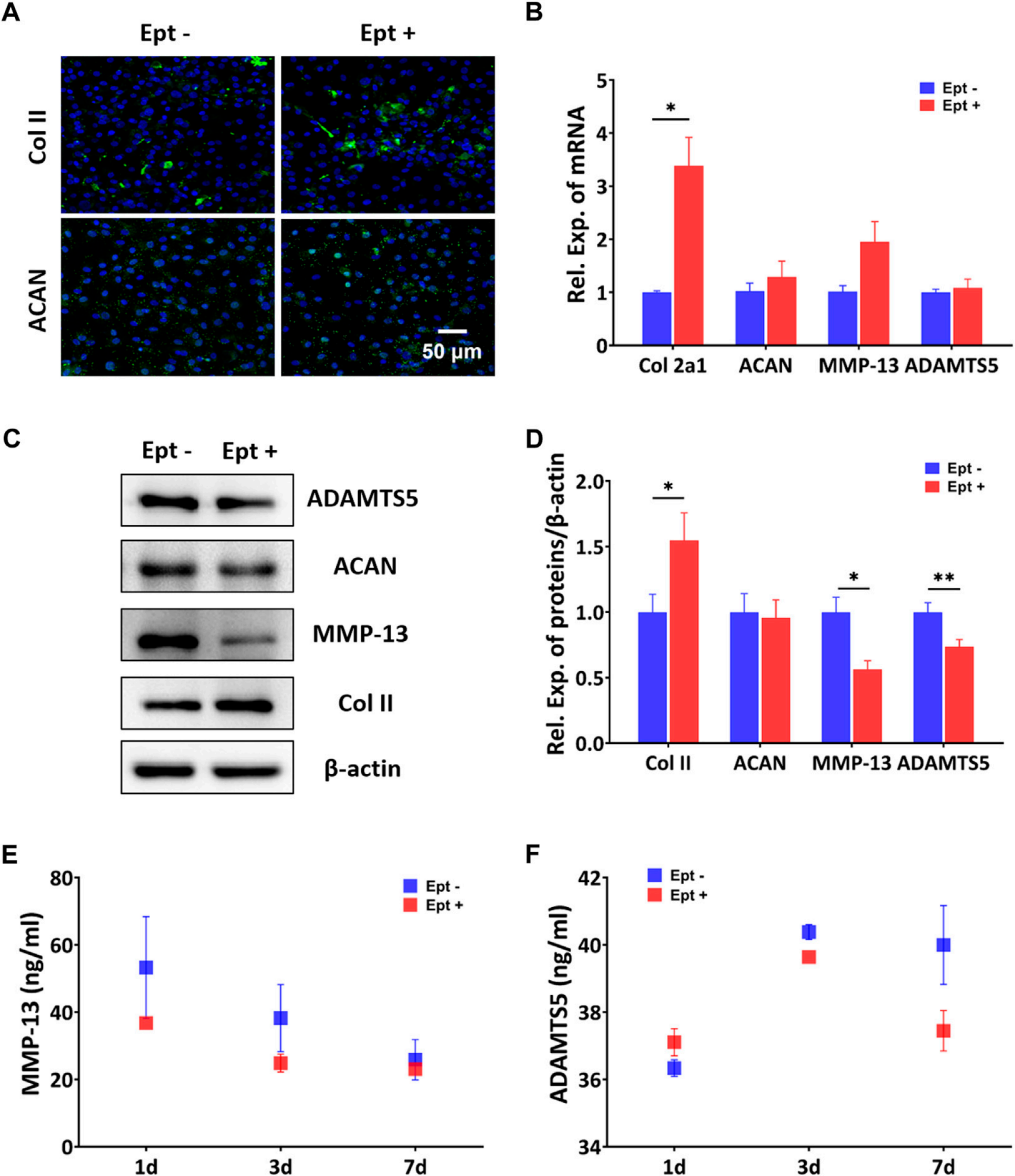
Regulatory effects of Ept on catabolic and anabolic dynamics in hOACs. **(A)** Immunofluorescent staining against Col II and aggrecan (ACAN) in Ept or vehicle treated hOACs. **(B)** PCR results of Col II, ACAN, MMP-13, and ADAMTS5 (*n* = 3). **(C)** Representative western blot detection of ADAMTS5, ACAN, MMP-13, Col II, and β-actin (*n* = 3). **(D)** Normalized quantitative data from western blot assay in Ept or vehicle treated hOACs. **(E and F)** Elisa detection of ADAMTS5 and MMP-13 levels in culture medium (n = 3). Mean ± SD, *p* ^*^ < 0.05, *p* ^**^ < 0.01.

CCK-8 assay was used to screen the optimal dose of Ept for culturing hOACs and hBMSCs. After culturing hOACs or hBMSCs with different concentration of Ept (0 μg/ml, 5 μg/ml, 10 μg/ml, 20 μg/ml, and 40 μg/ml) for 48h, the morphological differences of the cells were observed under optical microscope (Figure 3A). For CCK-8 assay, the OD value of hOACs and hBMSCs treated with 40 μg/ml Ept was significantly lower than those in the other groups (Figure 3B and C, n = 6). These observation indicated the inhibitory effect of Ept on viability of hOACs and hBMSCs at 40 μg/ml. Immunofluorescent staining was performed on type II collagen and ACAN after 48 h treatment of Ept in hOACs. As shown in Figure 4A, stronger fluorescence was detected in Ept treated group. Cartilage matrix anabolic genes Col2a1 and ACAN, and cartilage matrix catabolic genes MMP-13 and ADAMTS5 were detected by PCR. As shown in Figure 4B (n = 3), Ept significantly promoted Col2a1 gene expression (*p* = 0.011), and other three genes showed a slight increase tendency of expression (*p* > 0.05). In protein level, Ept significantly promoted type II collagen synthesis (Figure 4C; Figure 4D, n = 3, *p* = 0.018), whereas decreased ACAN (*p* = 0.15), MMP-13 (*p* = 0.012) and ADAMTS5 (*p* = 0.005) protein synthesis (Figures 4C,D). Furthermore, based on ELISA assay, level of MMP-13 in culture medium was down-regulated at day 1 and 3, and ADAMTS5 was down-regulated at day 3 and 7 post Ept treatment (Figures 4E,F, n = 3). As the secretion of MMP-13 and ADAMTS5 after synthesis in chondrocytes was regulated by external environment (Chao et al., 2011; Nam et al., 2016), Figure 4E showed different statistic difference, but shared the same tendency that Ept treatment decreased the expression of MMP-13 and ADAMTS5 in hOACs.

#### Influence of ept on hBMSCs chondrogenesis

**FIGURE 5:**
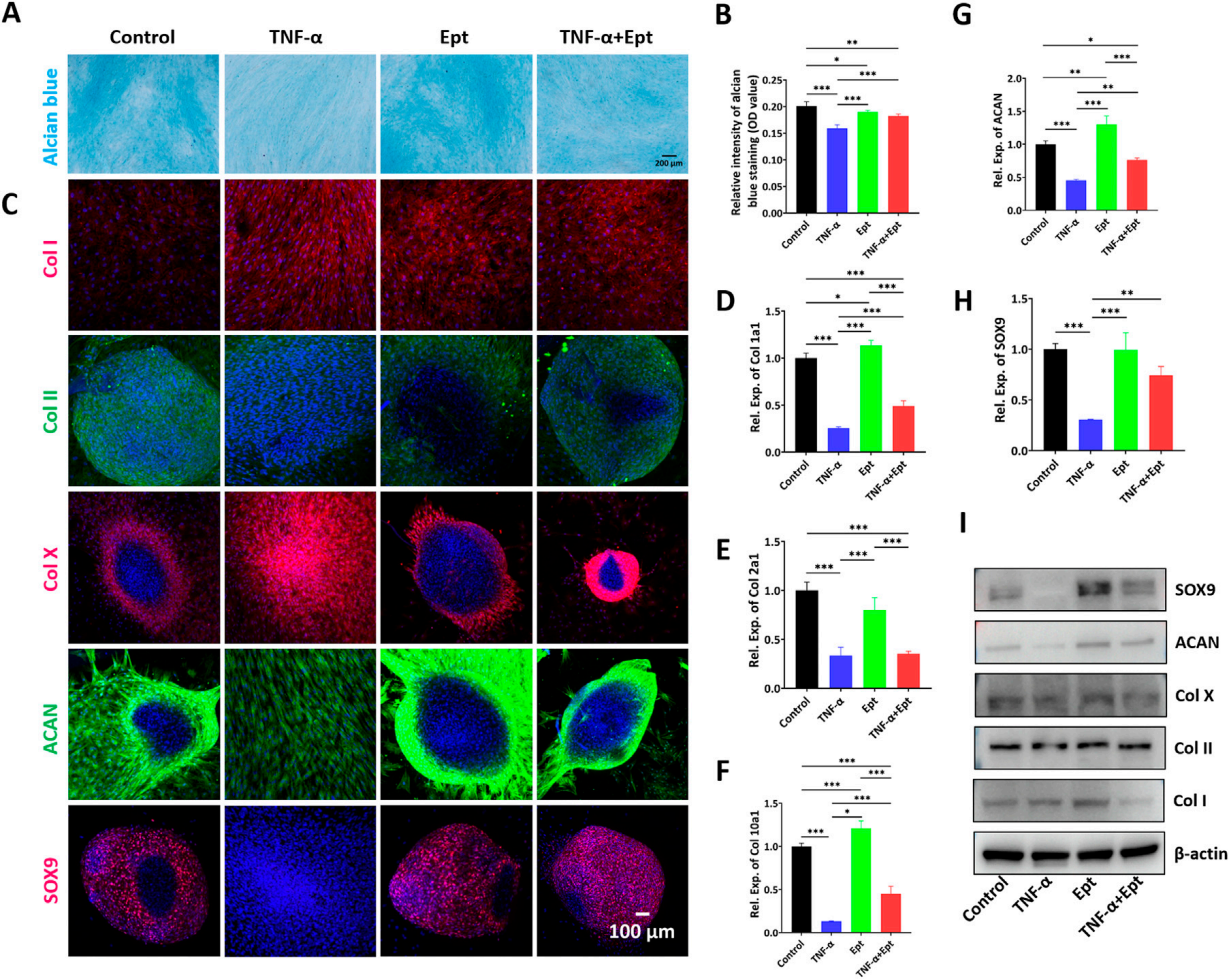
Effects of Ept on hBMSCs chondrogenesis. **(A,B)** Representative alcian blue staining and quantitative analysis (*n* = 5), **(C)** Representative immunofluorescence images of Col I (red color), Col II (green color), Col X (red color), ACAN (green color) and SOX9 (red color), **(D–H)** Relative mRNA expression of Col 1a1, Col 2a1, Col 10a1, ACAN and SOX9 (*n* = 3), **(I)** Representative western blot detection of Col I, Col II, Col X, ACAN and SOX9 in hBMSCs. Mean ± SD, *p* ^*^ < 0.05, *p* ^**^ < 0.01, *p* ^***^ < 0.001.

After chondrogenic inducing culture for 14 days, alcian blue staining was carried out to verify the cartilage matrix deposition in hBMSCs. As shown in Figure 5A, compared with control group, the TNF-α group showed the weakest staining intensity. Addition of Ept attenuated the negative effect of TNF-α on cartilage matrix deposition (Figure 5B). During 2-week chondrogeic induction, hBMSCs continuously secreted extracellular matrix, and spontaneously form pellet morphology. However, TNF-α inhibited the pellet formation in hBMSCs. The fluorescent intensity of type II collagen, ACAN and SOX9 was weaker in TNF-α group, while type I and type X collagen was stronger than the other three groups (Figure 5C). The chondrogenic markers were quantified by RT-qPCR (Figures 5D–H) and Western Blot (Figure 5I). In accordance with alcian blue and immunofluorescent stainings, the lowest gene expression of Col2a1, ACAN, and protein level of type II collagen, ACAN were observed in TNF-α group (Supplementary Figure S2). Ept attenuated the negative effect of TNF-α on chondrogenic markers. In addition, Ept alone promoted hBMSCs chondrogenic differentiation as Ept group showed the highest ACAN gene expression and type II collagen synthesis.

## Discussion

In the present study, the Ept embedded SF/PL hydrogel was fabricated and utilized to verify the influence of inflammatory environment on the articular cartilage regeneration induced by BMS. The addition of Ept into SF/PL hydrogel during BMS was proven to promote articular cartilage regeneration in a rabbit osteochondral defect model. *In vitro* study showed that Ept improved the cartilaginous matrix deposition and restrain catabolism of hOACs, as well as promoted chondrogenesis of hBMSCs in the presence of TNF-α.

Due to the limited intrinsic healing capacity of articular cartilage, surgical intervention such as BMS is required for treatment of focal articular lesions, osteochondritis dissecans, and degenerative cartilage lesions (van Eekeren et al., 2012). During BMS, drilling into the debrided chondral bone induces hematoma effusion containing BMSCs, growth factors, and cytokines at surgical site. Considering the insufficient mechanical property of blood colt, scaffolds were employed to stabilize the colt during BMS in both clinical and laboratory studies (Hoemann et al., 2005). To enhance the outcome of BMS, the previously fabricated SF/PL hydrogel served as bio-scaffold for both BMSCs adhesion and gradual release of Ept in this study. SF is a natural biopolymer extracted from Bombyx mori cocoons, has been regarded as one of the most promising candidates for tissue engineering and regenerative medicine due to its good biocompatibility, excellent mechanical strength, and slow degradation (Huang et al., 2018; Zhou et al., 2018). Pullulan is a neutral, biodegradable and non-toxic polysaccharide and widely used in biomedical applications (Singh et al., 2017). In our previous study, SF and tyramine substituted carboxymethylated pullulan were enzyme crosslinked. The compressive modulus of fabricated SF/PL hydrogel was 71.4 ± 9.3 kPa, which was in the range of that of osteochondral tissue. Slow degradation of the hydrogel in protease XIV solution retained porous microstructure, which enabled BMSCs ingrowth, chondrogenic differentiation and cartilage matrix deposition (Li et al., 2018).

The main purpose of this study was to evaluate the anti-inflammatory efficacy of Ept in promoting cartilage repair based on BMS. In order to simulate the BMS procedure, osteochondral defect in trochlear groove was created. The acute osteochondral injury induced by BMS significantly increased the expression of inflammatory cytokines in synovial fluid. Clinical evidence indicated that knees with osteochondral fracture had immediate increment in concentrations of TNF-α compared with knees without osteochondral fracture (Sward et al., 2014). During BMS procedure, bone marrow effusion, along with damage associated molecular patterns (DAMPs) that arose from tissue injury were released into articular cavity. Proinflammatory cytokines involved in traumatic reaction, such as IL-1β and TNF-α produced by chondrocytes, mononuclear cells, osteoblasts and synovial tissues, induce the production of a number of inflammatory and catabolic factors (Sahu et al., 2019). In a recent study, microfracture failure was found to have a positive correlation with TNF-α revealed by correlation analyses between the osteoarthritis research society international (OARSI) total score and the cytokines measurement (Danilkowicz et al., 2021). In our study, the ICRS Gross Scoring and the ICRS Histological Scoring results confirmed that addition of Ept in SF/PL hydrogel was beneficial to cartilage regeneration. To mimic the clinical practice, the concentration of Ept in SF/PL hydrogel was decided by converting equivalent dosage between rabbit and human according to body surface area. As the recommended dosage of Ept for adult is 50 mg/week, about 4 mg of Ept was needed for rabbit in the present study. The release experiment showed that the fabricated SF/PL hydrogel could continuously release about 85% of the embedded Ept in 5 days (Figure S1), which means the scaffold design (4 mg of Ept embedded in 200 ml SF/PL hydrogel) is suitable to antagonize the chronic proinflammatory effect of TNF-α in our rabbit model. Detailed histological analysis revealed that the cellular density in the regenerated tissue was higher in Ept + group, and most cells were spatially distributed in the newly formed lacuna-like structure. Moreover, as evidenced by safranin-O and IHC against type II collagen data, the newly formed hyaline cartilage tissue was seamlessly integrated with adjacent native tissue in the Ept + group, indicating that Ept might inhibit catabolism and promote anabolism of chondrocytes in an acute injury induced inflammatory microenvironment.

The negative effects of catabolic cytokines on cartilage have been well documented. Elevated levels of IL-6 and TNF-α in the injured cartilage were implicated in the IL-6 and TNF-α-mediated cartilage degradation (Sahu et al., 2019). To further illustrate the influence of TNF-α on BMS induced cartilage regeneration, the effect of Ept on chondrocyte phenotype, and on hBMSCs chondrognesis were assessed *in vitro*. Our data indicated that Ept treatment significantly promoted both gene and protein expression of type II collagen in hOACs, while decreased the protein levels of MMP-13 and ADAMTS5. The regulatory effect of Ept between catabolic and anabolic dynamics in chondrocyte might explain the superior integration in Ept + group from *in vivo* observation. The regeneration of articular cartilage induced by BMS was attributed to BMSCs chondrogenesis. Instead of chondrocytes migrated from adjacent articular cartilage, autoradiography using 3 H-thymidine and 3 H-cytidine showed that the repair was achieved by the differentiation of mesenchymal cells from the underlying bone marrow (Shapiro et al., 1993). Hence, the addition of Ept might have a positive effect on BMSCs chondrogenesis in the inflammatory microenvironment. In accordance with previous study (Heldens et al., 2012), TNF-α was found to inhibit hBMSCs chondrogenesis in control group. Whereas, addition of Ept significantly reversed the negative impact of TNF-α, as illustrated by alcian blue staining, type II collagen and aggrecan stainings. These results were complied with histological data, in which positive and uniform deposition of type II collagen was noticed in the Ept + group alone. However, significant increase in both type I and II collagen post Ept treatment alone was noticed in our data. As evidenced by previous study (Wu et al., 2021), this contradiction between gene and protein levels indicated posttranscriptional modification of these genes during chondrogenetic differentiation. In addition, we noticed that the spatial distribution of hypertrophic marker, type X collagen was different among groups. Although the gene expression level of type X collagen was elevated after Ept treatment (Figure 5F and I), the distribution was mainly in the peripheral area of deposited ECM in untreated and Ept treated hBMSCs. This observation along with previous studies (Knuth et al., 2019; Côrtes et al., 2021) suggested that ECM remolding by type X collagen was potentially required during hyaline cartilage matrix expansion (Figure 5C, COL II & ACAN).

## Conclusion

In the present study, SF/PL hydrogel containing Ept was fabricated, and the synergistical effect of SF/PL hydrogel and Ept was verified by BMS simulation. The addition of Ept into SF/PL hydrogel was proven to promote articular cartilage regeneration *in vivo*, potentially attributed to regulation of catabolic and anabolic dynamics in adjacent chondrocytes and enhancement of BMSCs chondrogenesis. Our finding might provide novel strategy for BMS augmentation, and expand the indication of current pharmaceuticals.

## Data Availability

The original contributions presented in the study are included in the article/Supplementary Materials, further inquiries can be directed to the corresponding authors.
