# Supplementary material for: Etanercept embedded silk fibroin/pullulan hydrogel enhance cartilage repair in bone marrow stimulation
Source: Front Bioeng Biotechnol. 2022 Dec 8;10:982894. doi: 10.3389/fbioe.2022.982894 (PMC9772014; doi:10.3389/fbioe.2022.982894)

Supplementary Material

1.1 Supplementary Figures

Figure legends

Figure.S1. The release curve of the etanercept in the hydrogel in vitro. The abscissa axis represents the time point of detection, and the vertical axis represents the released percent of etanercept.

Figure.S2. Normalized quantitative data from western blot assay of Col I, Col II, Col X, ACAN and SOX9 in hBMSCs.

Figure.S1.

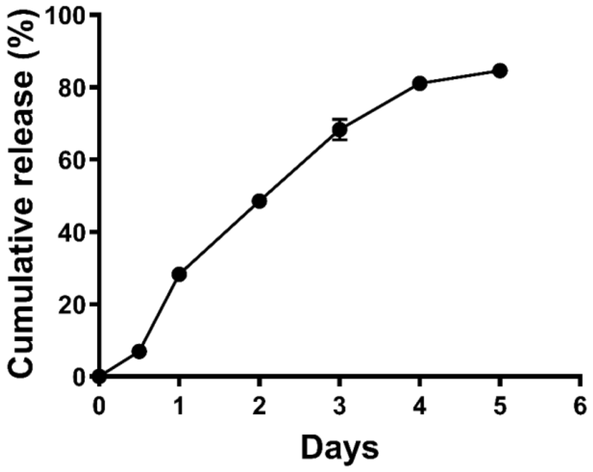

Figure.S2.

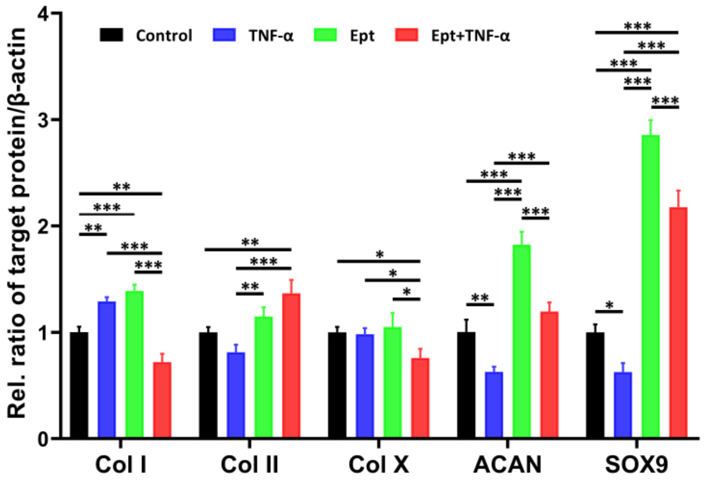

Supplement: Supplementary file 1 [file DataSheet1.pdf]
